# Seeing Keratinocyte Proteins through the Looking Glass of Intrinsic Disorder

**DOI:** 10.3390/ijms22157912

**Published:** 2021-07-24

**Authors:** Rambon Shamilov, Victoria L. Robinson, Brian J. Aneskievich

**Affiliations:** 1 Graduate Program in Pharmacology & Toxicology, Department of Pharmaceutical Sciences, University of Connecticut, 69 North Eagleville Road, Storrs, CT 06269, USA; rambon.shamilov@uconn.edu; 2 Department of Molecular and Cellular Biology, College of Liberal Arts & Sciences, University of Connecticut, 91 North Eagleville Road, Storrs, CT 06269, USA; victoria.robinson@uconn.edu; 3 Department of Pharmaceutical Sciences, School of Pharmacy, University of Connecticut, Storrs, CT 06269, USA

**Keywords:** filaggrin, keratinocyte, liquid–liquid phase separation (LLPS), loricrin, proteinaceous membraneless organelle (PMLO), intrinsically disordered protein (IDP)

## Abstract

Epidermal keratinocyte proteins include many with an eccentric amino acid content (compositional bias), atypical ultrastructural fate (built-in protease sensitivity), or assembly visible at the light microscope level (cytoplasmic granules). However, when considered through the looking glass of intrinsic disorder (ID), these apparent oddities seem quite expected. Keratinocyte proteins with highly repetitive motifs are of low complexity but high adaptation, providing polymers (e.g., profilaggrin) for proteolysis into bioactive derivatives, or monomers (e.g., loricrin) repeatedly cross-linked to self and other proteins to shield underlying tissue. Keratohyalin granules developing from liquid–liquid phase separation (LLPS) show that unique biomolecular condensates (BMC) and proteinaceous membraneless organelles (PMLO) occur in these highly customized cells. We conducted bioinformatic and in silico assessments of representative keratinocyte differentiation-dependent proteins. This was conducted in the context of them having demonstrated potential ID with the prospect of that characteristic driving formation of distinctive keratinocyte structures. Intriguingly, while ID is characteristic of many of these proteins, it does not appear to guarantee LLPS, nor is it required for incorporation into certain keratinocyte protein condensates. Further examination of keratinocyte-specific proteins will provide variations in the theme of PMLO, possibly recognizing new BMC for advancements in understanding intrinsically disordered proteins as reflected by keratinocyte biology.

## 1. Introduction

### 1.1. Protein Intrinsic Disorder and Keratinocyte Biology

In silico, in vitro, and in vivo analyses of intrinsically disordered proteins (IDPs) are expanding the appreciation [1,2] of their diverse and multiple roles as “hubs” or foci of intracellular signaling, as scaffolds for protein condensates, and in contributing to other distinctive functions because of their presence as a “conformational ensemble” rather than one fixed three-dimensional structure [3]. This unique conformational characteristic is a consequence of amino acid sequences featuring a biased residue composition with increased polar, charged, and structure-breaking residues as compared to compaction-supporting hydrophobic residues. With increased structural flexibility, IDPs feature properties distinct from fixed three-dimensional structures including increased regulation by post-translational modification and greater promiscuity for binding partners. It is these partner proteins, then, that often confer a specific structural conformation to IDPs via their protein–protein interaction [3]. Despite the importance of signaling hubs and protein scaffolds in the cell biology and differentiation of keratinocytes, we found few reports of purposeful IDP investigations in these cells or, more broadly, in epidermal biology. This apparent disconnect, especially in light of what IDP characteristics could mean for interpreting the function of keratinocyte-specific and keratinocyte-expressed proteins, led us to initiate a synergy of IDP and epidermal keratinocyte biology to fill this knowledge gap. Our premise is that specific investigation of intrinsic disorder (ID) in keratinocyte-expressed proteins could facilitate the understanding of their function in ways heretofore not considered. Our approach utilized two complementary avenues: (i) an overview of the keratinocyte literature for IDP-relevant reports, and (ii) an extensive targeted bioinformatic assessment of ID in keratinocyte proteins, especially for those proteins contributing to keratinocyte-unique structures. We aimed to establish relevance for more extensive IDP-directed studies in cutaneous research and, at the same time, inform IDP researchers regarding the opportunities for discovery in the specialized tissue of the epidermis.

### 1.2. Epidermal Specialization and Keratinocyte-Related Protein Intrinsic Disorder

**Figure 1:**
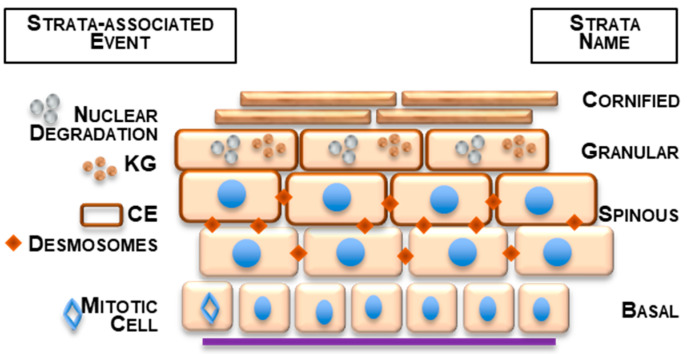
Simplified schematic of keratinocyte stratification in the human epidermis. KG indicates SFTP synthesis and keratohyalin granule formation. CE indicates cornified envelope protein synthesis and assembly. Desmosomes indicate numerous cell–cell contacts (“spines”) formed. Strata labeled on the right. Basal keratinocytes attach to the specialized connective tissue sheet, the basal lamina, at the bottom of the diagram.

The epidermis is the upper-most compartment of the skin. Due to the carefully regulated, progressive maturation of its major cell type, the keratinocyte, the epidermis provides an essential barrier function to protect the underlying dermis and deeper tissues [4]. Within the epidermis (Figure 1), keratinocytes form multiple layers (strata). For the epidermis in humans and other mammals, these are classically recognized as the basal layer, mitotically active and in direct contact with the underlying dermis; then, the spinous layer, post-mitotic keratinocytes with numerous cell–cell adhesion points (desmosome “spines”); the granular layer characterized by keratohyalin aggregates involved in keratin intermediate filament reorganization; and, ultimately, the cornified layer, exposed to the environment and composed of flattened keratinocytes (squames) for which nuclear degradation and extensive protein–protein cross-linking have taken place in their transition from granular to cornified (Figure 1). These layers are recognized by their histological position and specific proteins derived from strata- and keratinocyte-specific gene expression from basal to spinous to granular cells. Due to their key roles in normal cell physiology and certain disease states, the tertiary structure has been established for many of these strata-specific proteins. However, as we develop below, unstructured proteins, or IDPs, are likely to play significant roles in keratinocyte biology, and upon close inspection, many well-known keratinocyte proteins mirror the characteristics of IDPs.

As a representative of a comprehensive database, we queried PubMed for IDP reports relevant to keratinocyte biology (Table 1). Search results showed IDP characteristics in a diversity of several skin-associated proteins, but only a few investigations to date for keratinocyte-specific proteins. Considering the occurrence of IDPs across other cell types, we expect this is more reflective of dedicated IDP-type investigations targeting keratinocyte-specific proteins, having just in the last few years been reported, rather than keratinocytes, being particularly IDP-deficient. Nevertheless, the impact of intrinsic disorder from the other reports strongly predicts the significance for overall cutaneous biology, even if it is currently an under-investigated field. Although limited in the total number, the database hits revealed ID in diverse instances (Table 1) such as the following:

- Proteins of infecting bacterial and viral pathogens, the latter notably highlighting HPV oncoproteins;
- The dermal extracellular matrix protein elastin;
- Subdomains of familiar keratinocyte proteins, e.g., EGF receptor C-terminus and keratin N- and C-termini, for mediating protein–protein interactions in signaling and structural assembly, respectively;
- Specializations of non-human skin proteins for bio-reflectance or protection via skin-associated toxins in other organisms.

Publications on hornerin, BP180, and filaggrin [5,6,7] did, individually, make some direct mention of ID in keratinocyte-specific proteins and helped to support a broader consideration, as we report here. Hornerin’s repeat subdomains are extensively proteolytically processed in the skin to liberate antimicrobial peptides. Enhanced protease sensitivity, at least in vitro, is an established IDP characteristic [3]. The intracellular domain of BP180 was characterized as an ID region (IDR) using circular dichroism spectroscopy and amino acid content bioinformatics. Protein flexibility presumed to be conferred by ID within the BP180 intracellular domain may impact its interaction with other cytoplasmic-side proteins of the hemidesmosome, a keratinocyte cell–extracellular matrix attachment assembly. Lastly, filaggrin, as with hornerin, has extensive amino acid motif repeats, and, similarly, it undergoes extensive in situ proteolytic processing. However, unlike hornerin, products released from the filaggrin protein are associated with cutaneous hydration [8]. The contribution of ID to proteinaceous membraneless organelles, in part derived from liquid–liquid phase transitions [9], likely facilitates filaggrin’s participation in keratohyalin granules [7]. Even with these recent findings, a holistic IDP inquiry for keratinocytes, despite the dramatic potential to add to our understanding of the cutaneous protein function in health and disease, has yet to receive extensive research efforts.

### 1.3. Proteins Encoded by Genes of the Human EDC Are Enriched for ID Traits

**Figure 2:**
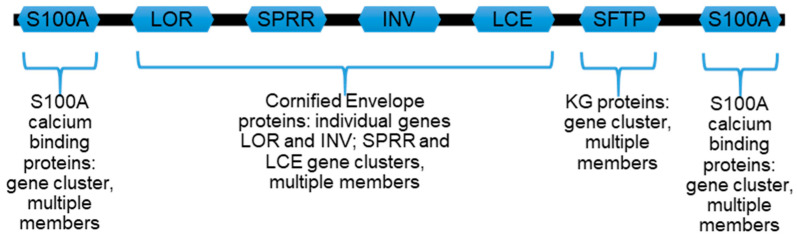
Schematic representation of human epidermal differentiation complex (EDC) on chromosome 1q21. The EDC is contiguous, as shown, but the chromosome region and genes/gene families are not to scale. S100A calcium-binding protein genes in up- and downstream clusters are different members of that family. CE, cornified envelope gene proteins: LOR, loricrin; SPRR, small proline-rich region (SPRR) proteins including cornifin-A and B; INV, involucrin; LCE, late cornified envelope proteins. KG, keratohyalin granule S100-fused type proteins (SFTPs): PF1, profilaggrin; HRNR, hornerin; PF2, profilaggrin 2; RPTN, repetin; CRNN, cornulin; TCHH, trichohyalin; TCHHL1, trichohyalin-like 1.

The epidermal differentiation complex (EDC) on human chromosome 1q21 contains over 60 individual genes (Figure 2) encoding keratinocyte maturation-dependent genes whose protein products provide characteristic structural and functional proteins of stratified squamous epithelia, such as the epidermis [40,41,42]. It is organizationally conserved across many mammalian and some other vertebrate species [43,44].

As an example of the EDC in general, the locus on human chromosome 1 (Figure 2) can be organized into major areas encoding the following: 

(i) A subfamily of seventeen S100 (S100A) calcium-binding proteins with different members flanking the EDC. 

(ii) Individual genes for loricrin and involucrin proteins which flank a related 11-gene subfamily for small proline-rich region (SPRR) proteins including cornifin-A and B. 

(iii) A distinct and extensive 18-member subfamily of additional late cornified envelope (LCE) proteins which, together with loricrin, involucrin, and SPRR, contribute to the cornified envelope (CE). The CE is a layer of proteins assembled under the cell membrane of later maturation stage keratinocytes (Figure 1) and is cross-linked by transglutaminases into a chemically resistant and detergent-insoluble “involucrum” (Latin, envelope).

(iv) Profilaggrin and its related S100 fused-type proteins (SFTPs) profilaggrin 2, trichohyalin, trichohyalin-like protein 1, repetin, hornerin, and cornulin, notable for their amino terminus S100-like calcium-binding EF hand domains [45], followed by (i.e., fused to) highly variable lengths of proteins often with repeating amino acid sequences.

Biomolecular condensates, brought about by LLPS and, in part, influenced by the protein’s intrinsic disorder, are currently recognized as droplets, granules, and speckles mediating diverse cell physiological processes and organizational events [46]. They can serve as nucleation points for self and partner proteins to associate despite low-affinity interactions leading to a spectrum of cellular consequences, e.g., stress response, increased activity efficiency, and formation of organizational hubs for other downstream assemblies [47,48,49,50,51,52]. Our work on the repression of keratinocyte intracellular inflammatory signaling by the intrinsically disordered protein TNIP1 [53], and the recent report of the keratinocyte ultrastructural protein profilaggrin as an IDP subject to LLPS [7] prompted us to actively examine other keratinocyte-expressed proteins regarding potential intrinsic disorder and possible phase separation given that much of keratinocyte differentiation is about maturation-dependent ultrastructural changes encoded by proteins from a unique genomic region known as the epidermal differentiation complex.

In the remaining sections, we consider ID traits for proteins representative of the human EDC. We do so with a view as to how such traits may contribute to the expected function of these late differentiation proteins, especially in light of ID contributing to scaffolding/hub functions or LLPS events, e.g., the EDC profilaggrin protein undergoing phase separation for keratohyalin granule formation, a quintessential histological feature of properly maturing human epidermal keratinocytes. Given that the EDC gene cluster is shared across mammalian and, to some extent, non-mammalian vertebrates, we ask if human protein ID characteristics, e.g., those as central to ID as compositional bias and repeat amino acid motifs, are present in other species, and what consequences may arise in LLPS from variations in ID thematic traits.

Here, we sought to synergize bioinformatic assessment of keratinocyte EDC proteins’ amino acid sequences in the contexts of ID and LLPS with how that might further explain processing and/or assembly in the setting of keratinocyte differentiation which is reliant upon several cell-specific proteins. In doing so, we can now newly propose (1) the addition of sheath-like or planar PMLO organizations from LLPS, (2) why the expression of sequence-related, intrinsically disordered “filament-aggregating proteins” across different species does not share similar intracellular granule formation, and (3) the wealth of discoveries that lie ahead from future studies of combinatorial bioinformatics and biophysical studies of keratinocyte-specific proteins revealing not only a better understanding of their cell-specific function but possibly new insights into ID and LLPS for other cell types.

## 2. Bioinformatic Evaluations of Keratinocyte-Specific Proteins from the EDC Locus

### 2.1. Assessing Protein Intrinsic Disorder Encoded in the EDC 

#### 2.1.1. S100A Proteins

S100A proteins from the EDC (Figure 2) are a family of small proteins (~10 kDa) with two EF-hand calcium-binding subdomains. Some (e.g., psoriasin, also referred to as S100A7) are over-expressed in or otherwise particularly associated (e.g., koebnersin, also referred to as S100A15) with hyperproliferative psoriatic keratinocytes [54]. Nevertheless, roles for S100 proteins beyond binding calcium or other divalent cations are incompletely defined [55]. We assessed three members (UniProt P23297, S100-A1; P31151, S100A7 psoriasin; and Q86SG5, S100A15 koebnersin) using PONDR-FIT, a meta-predictor of intrinsic disorder which provides a disorder score per amino acid residue with values greater than 0.5 being indicative of disorder [56]. PONDR-FIT presents a disorder score which is a combined output of multiple individual algorithms, trained on literature-described disordered proteins, which consider net charge, amino acid composition, hydrophobicity, and potential for inter-residue interaction across a protein length. For S100-A1, S100A7, and S100A15, we found global disorder scores, with average PONDR-FIT scores of all amino acids along the protein (see Methodology) of 0.422, 0.454, and 0.383, respectively. These are overall relatively lower compared to other EDC members reported below. These full-length S100A-type protein scores are consistent with the S100-like amino terminus of S100 fused-type proteins (SFTPs) such as profilaggrin. Its N-terminal amino acids 1-93 return a PONDR-FIT score of 0.445 compared to a score of 0.895 for the whole profilaggrin protein, and 0.905 for amino acids 94-4061. These similar scores of the S100A proteins’ and the SFTP N-termini are in keeping with an S100A-type ancestral gene giving rise to SFTPs through either fusions or possibly extension [57]. We included this brief mention of S100A proteins for completeness as they are part of the EDC, and due to their likely relationship to S100 fused-type proteins. However, with their typically moderate intrinsic disorder scores relative to other EDC members, they will not be considered further in this report.

#### 2.1.2. Loricrin, Involucrin, SPRR, and LCE

**Figure 3:**
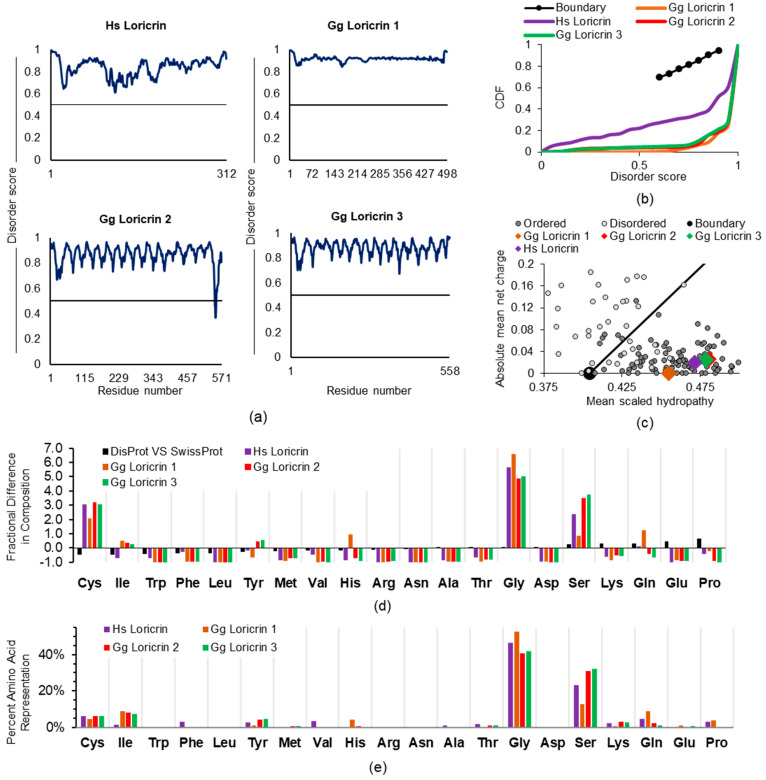
ID analysis of loricrin, a major cornified envelope protein. (**a**) Meta-predictor-driven evidence of intrinsic disorder in human (*Homo sapiens*, Hs) and 3 forms of chicken (*Gallus gallus*, Gg) loricrin. Boundary line at 0.5 segregates disordered (above 0.5) amino acid residues from ordered (below 0.5). (**b**) Cumulative distribution function (CDF) distinguishes ordered proteins from disordered proteins with increased predicted disorder content driving the plotted points below the boundary (black dotted) line. (**c**) Charge–hydropathy (CH) plot describes intrinsic disorder likelihood by evaluation of absolute mean net charge versus the mean scaled hydropathy of a protein. Ordered standards (light gray circles) and disordered standards (dark circles) provide context for predicted scores of evaluated proteins (various colored diamonds). (**d**) Abundance of amino acids evaluated against those determined for proteins contained in the DisProt database versus the SwissProt database (black bar). (**e**) Amino acid abundancies presented as total percentage of total amino acid content.

The loricrin protein comprises ~70% of the fully matured epidermal keratinocyte CE structure compared to involucrin’s contribution of ~3% [42]. Thus, it is a major component of the cross-linked proteins comprising the sheath-like layer under the cell membrane of late differentiation keratinocytes. As with involucrin, which we will detail elsewhere, our computational results with loricrin (Figure 3), such as a 0.840 PONDR-FIT score average along the entire protein, with no residue < 0.5, are entirely consistent with initial loricrin biophysical publications and conformational interpretations. Although not referred to as intrinsic disorder per se, a deep dive into the loricrin literature shows an early expectation of a “little organized structure” because of its glycine repeats [58]. Loricrin is repeatedly described to have flexible qualities [59,60] which could be expected to confer malleability to at least the early stages of CE formation, thus facilitating protein access for other CE constituents to the developing structure.

Coding sequences and expression of the EDC member and CE protein loricrin have been reported across several mammalian and non-mammalian species [43,61]. We find that the human (*Homo sapiens*, Hs) loricrin and the published three chicken (*Gallus gallus*, Gg) homologues all share, for their full-length proteins (Figure 3a), a very high average disorder score of ≥ 0.840 (PONDR-FIT global score: Hs 0.840, Gg1 0.921, Gg2 0.864, Gg3 0.878). This is visualized as a cumulative distribution function (CDF) (Figure 3b), where these proteins are shown to have an increased distribution of disordered residue scores, resulting in a concave plot, well below the boundary line, as is typical for IDPs. This in-common high disorder score for the four sequences was present despite that, across human and chicken loricrin proteins, we find a shared, but unexpected, enrichment for the order-promoting amino acid cysteine. This unique feature is apparent looking at the charge–hydropathy (CH) plot, where disordered proteins [62] are found left of the boundary, among the more charged, less hydrophobic proteins (Figure 3c). Although all other in silico methods indicate these proteins are disordered in solution, they are among the ordered proteins in the CH plot. The increased occurrence of hydrophobic cysteines may, in part, explain this phenomenon. During incorporation of loricrin into the developing CE late in keratinocyte differentiation, it participates in extensive disulfide bond formation in addition to intra- and inter-protein cross-links made by transglutaminases [58,63]. A comparison of all proteins of the DisProt (disordered proteins, D) versus SwissProt (ordered proteins, O) reference databases [64] returns a reduced occurrence of cysteine (Figure 3d) in disordered proteins [(D-O)/O; −0.47]. In contrast, these four loricrin sequences average a +2.85-fold enriched occurrence for cysteine (Figure 3d). The cysteine order-promoting effect may be reduced by the cumulative effect of other residues, thus maintaining an overall high disorder score. For instance, the four full-length loricrin sequences also share enrichment for the disorder-promoting [65] residues glycine and serine (averaging about +5.54 and +2.62, respectively), at proportions many times higher than the fold preference present for the same two amino acids (glycine, +0.06; serine, +0.27) across all proteins of the DisProt and SwissProt databases (Figure 3d). It may be that the increases in glycine and serine disorder-promoting residues compensate for this cysteine enrichment. For instance, glycine constitutes > 40% and serine up to 32% of all residues in these loricrin proteins (Figure 3e). This needed approach for holistic assessment of ID in loricrin is also reflected in the apparent discrepancy in the CDF and CH plots (Figure 3b,c). CH can be skewed by the amino acid content, even for legitimate IDPs. In contrast, CDF looks more broadly at predictive disorder scores across protein amino acid contents, and this reflects IDPs based on more global assessments such as PONDR-FIT.

In regard to the possible loricrin participation in LLPS, it is fascinating to consider the >30-year-old description by Steven et al. [66] of loricrin immuno-detection in mouse skin at the electron microscope level. They reported the loricrin protein as the “first accumulated in a particular class of cytoplasmic granules” (distinct in size, shape, and protein content from filaggrin-containing keratohyalin granules) which, at a late and very transient stage of keratinocyte differentiation, distributes partially throughout the cytoplasm and then, ultimately, is “rapidly incorporated into the cornified cell envelope” at the cell perimeter. Their summation of these events as a “precursor–product relationship” addressed, for loricrin, the cell biology question of why “major proteins of terminally differentiated keratinocytes are first stockpiled in separate kinds of cytoplasmic granules” instead of “straightforward synthesis … at the designated step in the differentiation pathway”. They recognized that this “abrupt” step is a “transitional state” of late keratinocyte differentiation otherwise characterized by “diminished (mRNA and protein) biosynthetic competence” and suggested such compartmentalized protein stores would be advantageous for rapid “kinetic” remodeling of the cells made possible by proteins “presynthesized and stored until the appropriate time”. Such terminology for loricrin is completely compatible with the currently proposed advantages of IDP depots in membraneless organelles to “alter their internal equilibrium” [46], especially for repeat-rich and low-complexity IDPs [67], and with the overall characteristics of other highly disordered proteins found in various membraneless organelles [68].

Reminiscent of advances made by progressive versions of intrinsic disorder algorithms, first-generation protein phase separator predictors [2,69] for biological condensates are now providing computational avenues for in silico investigations of candidate transitioning proteins. Two among these, LARKS [70], discussed here for loricrin, and catGRANULE [71], employed below for SFTPs, particularly help to call out the need for further computational and biophysical analysis of keratinocyte-expressed proteins in regard to LLPS.

Eisenberg and colleagues [70] performed human proteome analysis to identify the top 400 proteins most enriched for low-complexity, aromatic-rich, kinked segments (LARKS). Amongst this remarkable dataset of high-scoring proteins are those widely expressed such as the chromatin-associated zinc finger protein GATAD1 with 21 reported LARKS. Most interesting to us, numerous proteins specific to keratinocytes such as several of the LCE group (see above) were identified for their relatively high frequency of LARKS (e.g., UniProt Q5T751, LCE 1C, 28 LARKS; Q5TCM9, LCE 5A, 22 LARKS). The major CE component loricrin has over 90 qualifying peptide sequences within it for recognition as LARKS. Repeated, low-complexity domains containing LARKS, as termed by the authors [70], are the “Velcro” for assembling membraneless organelles, formation of which may be concentration-dependent and transient. This functional visualization along with the occurrence of LARKS in “proteins that may form networks and gels by multivalent interactions” seems to have been defined with the cornified envelope proteins loricrin and LCE in mind.

Within the human EDC (Figure 2), coding regions for loricrin and involucrin are separated by genes for additional CE components, the 11-small proline-rich region (SPRR) proteins [41,42]. Centromeric to involucrin are genes for the next subfamily, the LCE proteins. Our analysis indicates these relatively minor CE proteins from the SPRR and LCE families will also be characterized by extensive disorders. As with the major CE component loricrin, SPRR and LCE are enriched for order-associated cysteine (e.g., UniProt Q9BYE4 SPR2G, 15.1%; A0A183 LCE6A, 8.8%), but as expected from the SPRR (small proline-rich region) name, and also occurring in LCE, these two protein families show extensive inclusion of not only disorder-promoting proline (SPRR, 39.7%; LCE 13.8%) but also glutamine (SPRR, 13.7%; LCE 10.0%). While these trends establish ID as a likely CE protein trait, it does not seem to be an absolute requirement to join the CE protein club. Cornifelin (UniProt Q9BYD5), another CE constituent, but encoded on chromosome 19 outside the EDC [72,73], appears to be mostly ordered (global average PONDR-FIT score 0.278). Thus, ID in both major (loricrin) and lesser components (involucrin, SPRR, LCE) of the CE seems certainly compatible with their participation in the formation of that differentiation-dependent structure. We suggest that the conformational flexibility of the components, before their covalent cross-linking to self and other CE proteins [74] by transglutaminases, may facilitate incorporation of the diverse proteins found in the final CE. Thus, if the CE can be considered the product of macromolecular crowding, as seen for other IDP-enriched structures [75], then there is some tolerance in its recipe both for the amino acid content of individual IDPs and incorporation of non-IDPs.

#### 2.1.3. Profilaggrin and Related S100 Fused-Type Proteins (SFTPs)

**Figure 4:**
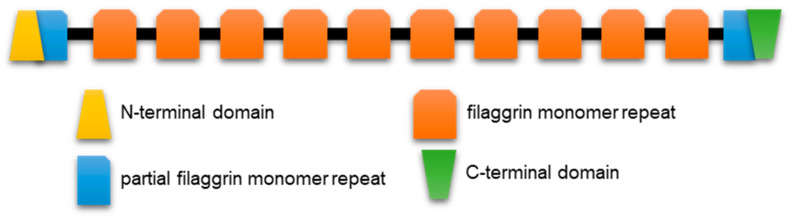
Protein schematic starting at the N-terminal domain (~290 amino acids) which includes sequence similarity to calcium-binding EF hands of the S100 protein family. Human profilaggrin has 10–12 complete repeats (~320 amino acids each) with linker regions of 19 amino acids. Mouse profilaggrin may have up to 16 repeats. C-terminal domain (~160 amino acids) sequences are required for proteolytic processing for monomer release.

Profilaggrin is the prototypical member of the related S100 fused-type proteins (SFTPs) which, in the human genome, include trichohyalin, trichohyalin-like protein 1, repetin, hornerin, profilaggrin 2, and cornulin. Human profilaggrin [8,76] is a high-molecular weight (> 400 kDa) polymer phosphoprotein with at least ten consecutive repeats from which the monomer filaggrin protein is derived (Figure 4). Profilaggrin, a major constituent of differentiation-dependent keratohyalin granules (KG), is ultimately dephosphorylated and proteolytically cleaved between the repeats to liberate filaggrin monomers through specific and carefully regulated steps. KG, eponymous of the epidermal granular layer (Figure 1), are easily seen at the light microscopy level, with routine histological staining reflecting the abundance of profilaggrin at this stage of keratinocyte specialization. Filaggrin monomers participate in the bundling of the keratinocyte’s namesake intermediate filament protein, keratin. Ultimately, the monomer filaggrin is proteolytically digested late in differentiation (granular-to-cornified layer transition, Figure 1), releasing hygroscopic free amino acids and their derivatives which contribute to skin hydration.

Filaggrin monomer release requires more than just cleavage sites between repeats. Mutations equating to absence of the usual carboxyl-terminus severely restrict monomer release, suggestive of some cis instruction from that region of the full-length polymer protein [8,76]. Loss-of-function mutations early in the coding sequence severely truncate the protein via the introduction of a stop codon in the first of the usual 10–12 repeats and lead to severe skin barrier disruption because of extensive epidermal flaking ([77] for review). There are small differences in the length and number of repeats across mammalian species, although this does not seem to negatively impact KG formation or keratin aggregation, as revealed in filaggrin’s name derivation from “filament-aggregating” protein.

Recently, in silico IDP traits of human profilaggrin were key to interpreting its contribution to the liquid–liquid phase separation of membraneless KG [7]. Hornerin, within the profilaggrin and related S100 fused-type protein group, has also been previously computationally described as an IDP [5] and a minor component of KG [78]. The unstructured conformations of IDPs are characteristically more accessible to proteases. This may promote profilaggrin proteolysis to its filaggrin monomer form. With hornerin [5], this may facilitate protease access to release cationic antimicrobial peptides derived from its numerous repeat sequences. As it might be expected from these genetically related sequences, other mammalian SFTPs share with profilaggrin a multiple repeat content, relatively low amino acid complexity, and some enrichment of disorder-promoting residues. From such traits, it is reasonable to expect these proteins, as recently reported for filaggrin [7], may be contributing to liquid–liquid phase separation driving KG formation.

While post-translational modifications such as phosphorylation could be expected to affect IDP performance, early work on recombinant filaggrin peptide phosphorylation reported granule formation was not dependent on phosphorylation [79], although extensive phosphorylation of the endogenous profilaggrin protein does occur. Importantly, the ability to establish direct and absolute conclusions on sequence content and post-translational processing, as they might affect KG formation, is limited. Many reports have examined recombinant filaggrin fragment contribution to KG. However, across them are differences in the length, number, and composition of individual repeats expressed for experimental studies as models of endogenous profilaggrin protein processing [7,79]. An investigative synergy of such variations along with mammalian profilaggrin phosphorylation and KG formation in light of liquid–liquid phase transition is warranted.

### 2.2. Examining Intrinsic Disorder in the EDC Proteins of Non-Human Species

#### 2.2.1. General EDC Protein Considerations across Species

The conservation and evolution of keratinocyte-expressed EDC gene sequences are intensely studied across species to investigate the roles of protein families (e.g., SFTPs) and individual proteins (e.g., CE protein loricrin) in generating a protective skin barrier function in the diverse environments inhabited by those species ([43,80] for review). Searching for EDC-like gene clusters in vertebrates has demonstrated at least partial homologues in amniotes (mammals, lizards, and avian) and amphibians, but not fish [43,57]. For instance, scaffoldin is an SFTP found in the EDC of avian and reptilian, but not mammalian, species [45]. From such a gene–familial relationship, we predicted and found that, even including the likely structured calcium-binding S100-type N-terminus, alligator scaffoldin displays the high disorder (PONDR-FIT score 0.817) characteristic of the SFTP group. Likewise, a GenBank inferred reference sequence (NP_001338424, 4295 aa) for chicken scaffoldin, which we retrieved via a search with a published partial 955 amino acid sequence [45], yields an equally high disorder (PONDR-FIT score 0.813), even with inclusion of the expected structured N-terminus. In addition to the SFTP ortholog presence or absence across species, gene representation in the EDC can also vary in number, as we presented above for the CE protein loricrin, with one gene in the human EDC, and three genes in the corresponding chicken locus.

The EDC gene set, which, as we show above, is enriched for IDPs in mammalian genomes, appears to have developed in parallel to vertebrate adaptation to a terrestrial environment [43,44,63], suggesting that the biochemical and biophysical characteristics of encoded proteins are advantageous for those surroundings. However, the gene clusters of the EDC, as introduced above, (i) S100A calcium-binding proteins, (ii) loricrin, involucrin, SPRR, and late cornified envelope proteins, and (iii) filaggrin and related SFTPs, are not all retained once having arisen in a class such as mammals. While dolphin sequences for involucrin and filaggrin have been reported, filaggrin is absent in whales. All other members of the SFTP genes and, likely, the LCE proteins have been lost in cetaceans (dolphins and whales) [81]. This suggests that if ID of these proteins was contributing to epidermal function, it, along with the protein, is dispensable for meeting barrier function in alternative environments or has been assumed by some other protein.

#### 2.2.2. SFTP Disorder: Frog Versus Human Sequence Considerations

In contrast to the human EDC, some non-mammalian correlates are more limited in the genes present, missing a subfamily entirely, or, for multi-gene families such as S100A- and SFTP-type groups, with only some of the members represented. The tetraploid nature of *Xenopus laevis* (Xl) [82] adds further opportunity for intra- and inter-species comparisons of ID of related proteins such as SFTPs. *X. laevis* has four differently sized SFTP genes, two each in its “L” and “S’’ subgenomes (chromosome 8S with SFTP1.S and SFTP2.S; chromosome 8L with SFTP1.L and SFTP2.L) [57]. These *Xenopus* SFTP sequences have the S100 fused-type protein organization found in other genomes but have not been associated with specific SFTP mammalian homologues (e.g., filaggrin or hornerin). Mlitz and colleagues [57] noted there are some extensive amino acid compositional differences such as ~2–5-fold less histidine in frog versus human SFTPs depending on which individual sequences are compared. Due to these amino acid differences, they suggested the expected proteolytic products from these frog proteins may not provide the same hydrating or antimicrobial functions as filaggrin and hornerin, respectively, in humans. We examined what consequences compositional differences might have on the predicted disorder. 

**Figure 5:**
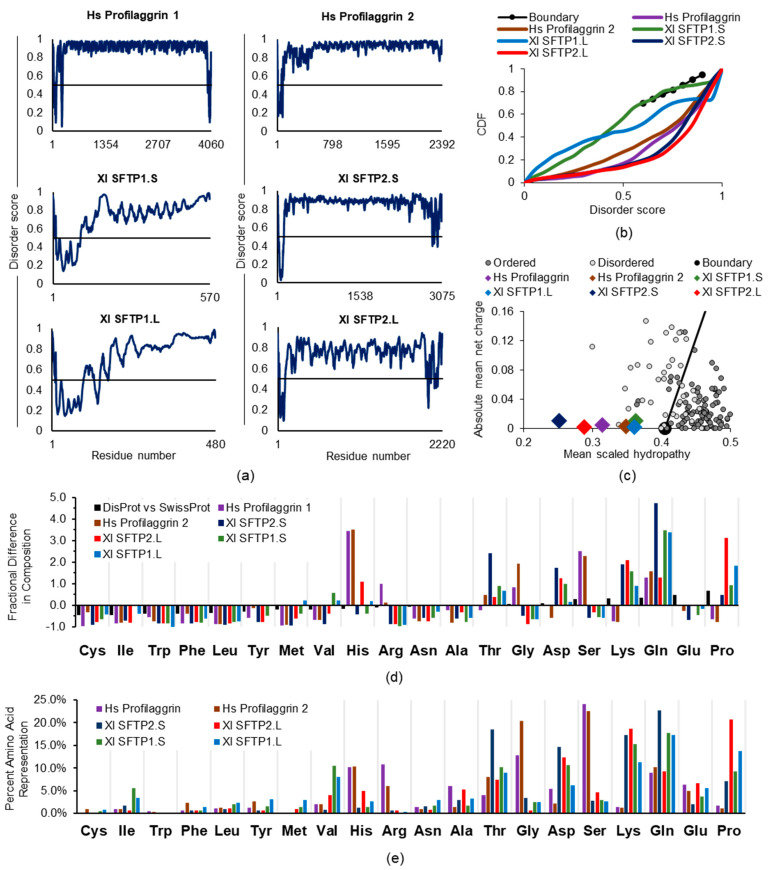
ID analysis of human profilaggrins 1 and 2, and four frog SFTPs. For explanations of panels (**a**) through (**e**), please refer to analysis as described in Figure 3’s legend. Human, *Homo sapiens*, Hs. Frog, *Xenopus laevis*, Xl.

Amino acid sequence identity between human profilaggrin and any of the four *Xenopus* SFTPs is limited: SFTP1.S (36%), SFTP2.S (34%), SFTP1.L (35%), and SFTP2.L (34%) [57]. There is some increase when other human SFTPs, which make lesser contributions to mammalian KG, are the point of comparison, e.g., human hornerin (UniProt Q86YZ3) and Xl SFTP2.L (41%). Based on the anticipated amino acid sequences from published [57] complete proteins (Xl SFTP1.S) and those inferred from *Xenopus* whole genome sequencing GenBank deposits (Xl SFTP1.L, XP_018087213.1; Xl SFTP2.S, OCT66701.1; Xl SFTP2.L, OCT69537.1), we determined that, as with the human SFTPs profilaggrin and profilaggrin 2, these frog proteins share a high predicted disorder (Figure 5a). This is especially apparent (Xl SFTP1.S, 0.790; Xl SFTP1.L, 0.790; Xl SFTP2.S, 0.869; Xl SFTP2.L, 0.757) carboxyl to the presumptive N-terminal S100 calcium-binding domain, suggesting that, despite the reduced identity from the amino acid sequence divergence, ID is a shared trait. This assessment is supported by five of the six SFTPs falling well below the boundary line in the CDF plot (Figure 5b), with just frog SFTP1.S overlapping that demarcation. Likewise, all six SFTP proteins are found left of the boundary in the CH plot (Figure 5c). Nevertheless, while ID is shared, these frog SFTPs may structurally perform in amphibian keratinocytes differently than their mammalian counterparts.

#### 2.2.3. SFTP Disorder and Liquid–Liquid Phase Separation: Frog Versus Human Sequence Evaluations

The human SFTP profilaggrin is the major component of KG in keratinocytes [77]. KG formation is dependent on liquid–liquid phase separation [7]. For human profilaggrin 1 (Hs PF1), we calculated (Table 2) a high disorder score (0.895 including the N-terminus) and a high arginine bias (0.885, calculated as ARG/[ARG + LYS]) across its large size (UniProt P20930, 4016 amino acids). Together, these traits and high serine content [65] possibly compensate for its histidine-enriched composition (10.2%), as suggested for other proteins [83] undergoing LLPS. Interestingly, IDPs enriched in arginine ([84] for review), such as the Hs PF1 and Hs PF2 (Table 2), have a greater tendency to undergo phase separation in contrast to those high in lysine, such as the frog SFTPs, consistent with predictions reported for other lysine-enriched proteins [85]. There is also evidence ([84] for review) histidine may contribute to LLPS assembly/disassembly for the relatively large human SFTPs profilaggrin and profilaggrin 2 (Table 2) if, as in mammalian keratinocytes, there are appropriate shifts in the intracellular pH [7].

Considering the shared IDP characteristics of human and frog SFTPs, but also their compositional differences, it is intriguing to note the easy detection of KG at the light microscope level in mammalian epidermis, but the KG absence [57] in frog skin. This occurs despite the presence of the four SFTPs, two of which, Xl SFTP 2.S and 2.L, are relatively large proteins, 3075 and 2220 amino acids, respectively, approaching the lengths of human profilaggrin and profilaggrin 2, at 4061 and 2391 amino acids, respectively. These two frog SFTPs exhibit high disorder scores (Xl SFTP 2.S, 0.852, and Xl SFTP 2.L, 0.740, calculated including the structured N-terminus). Notably, they (Table 2) have a histidine content (1.3 and 5.0%, respectively) and an arginine bias (0.031 and 0.032, respectively) inverted from human or other mammalian profilaggrin sequences. Additionally, and strikingly, the aliphatic index for the two smaller *Xenopus* SFTPs, 1.S and 1.L (570 and 480 residues, respectively), is 2.5–3-fold greater than that for human profilaggrin and profilaggrin 2. A high aliphatic index, which represents the relative volume occupied by side chains of alanine, valine, isoleucine, and leucine, positively correlates with hydrophobicity. While not formally referring to them as SFTPs, Alibardi [86] earlier reported on detecting small amounts of keratin filament-associated proteins via radiolabeling of frog skin. It was suggested their low amounts were insufficient to assemble KG, as seen in mammalian epidermis at the light microscope level.

Human profilaggrin and profilaggrin 2, along with the four frog SFTPs, are enriched for disorder-promoting amino acids, although the nature and proportion of these residues differ (Figure 5d,e). In addition to the histidine and arginine differences noted above, these two human SFTPs are also 7-fold higher in serine than the frog proteins, possibly adding significant conformational flexibility to the profilaggrin and profilaggrin 2 backbones, as has been proposed [65], as a consequence of serine enrichment. In contrast to these two human SFTPs, the frog SFTPs are enriched in asparagine, glutamine, and lysine, most commonly found on protein surfaces. While frog SFTPs also contain 5–10-fold more proline, which is ranked highest amongst the amino acids for disorder propensity [87], this quintessential IDP characteristic alone appears insufficient for phase separation to KG. In sum, despite certain IDP traits of frog SFTPs, their high content of charged disorder-promoting residues and other compositional characteristics (Table 2) may favor protein solubility rather than KG phase separation.

#### 2.2.4. SFTP Liquid–Liquid Phase Separation: Further In Silico Assessments

**Figure 6:**
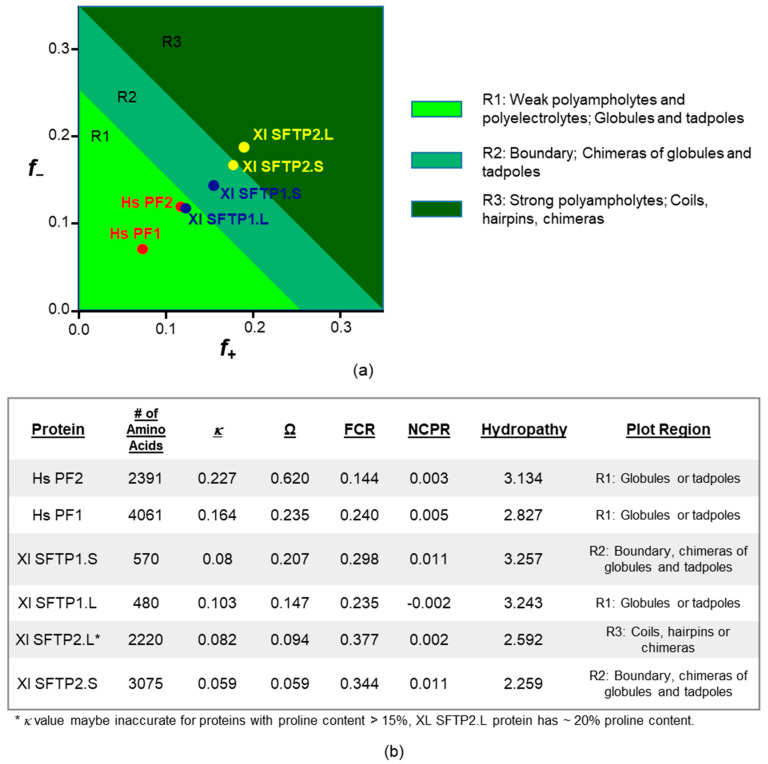
Output of CIDER analysis of human and *Xenopus* SFTP proteins. (**a**) Das–Pappu diagram of states depicting the proposed structural conformation for each protein based on the fraction of positive (*f*+) and negative (*f*−) charged residues. (**b**) Proteins are sorted in the table according to their Ω values. Human profilaggrin (Hs PF1) and filaggrin (Hs PF2) (red dots) have the lowest fraction of charged residues, placing them into the R1 region of the diagram, implying they adopt a globular or tadpole-like shape. The *Xenopus* SFTP 1.L and SFTP 1.S proteins (blue dots) fall into regions R1 and R2, respectively. Proteins that occupy region 2, or the boundary region, assume an ensemble of states between those of R1 and R3. The *Xenopus* SFTP 2.S and SFTP 2.L (yellow dots) lie adjacent to and firmly in R3, meaning they have the potential to fold into hairpin or coiled conformations. FCR, fraction charged residues; NCPR, net charge per residue.

Across frog and human SFTPs, it is interesting to note the retention of ID as an endpoint of the amino acid composition (Figure 5a,b), if it is not preservation of the exact same residue identities providing it (Figure 5d,e), a concept supported by proteome and protein family studies [88,89]. We queried the conformational variety [90] from such compositional differences for human profilaggrin and profilaggrin 2, along with the four frog SFTPs, via CIDER [91] for classification of intrinsically disordered ensemble relationships (Figure 6a,b). All of the proteins had net charge per residue (NCPR) values close to zero, well below the threshold value of 0.25, consistent with compact globular ensembles. Perhaps more telling are the differences, rather than group-wide similarities, of these disorder-sharing SFTPs. Human profilaggrin and profilaggrin 2 lie in region 1 (R1) of the plot, indicative of globule or tadpole formation (Figure 6a,b). Their κ and Ω values are notably larger than those calculated for the frog proteins. This reflects a segregation of proline and oppositely charged residues along their sequences, especially true for human profilaggrin 2, with an Ω value of 0.620. Additionally, Xl SFTP1.L is found in R1, with a larger fraction of charged residues (FCR) and lower κ and Ω values, which may possibly lead to a smaller radius of gyration. Xl SFTP1.S lies within region 2 (R2) of the plot and has an intermediate level and mixing of charged residues. Proteins within this boundary region are best described as ensembles or chimeras of globules and coiled conformations. The only protein in region 3 (R3) of the plot is Xl SFTP2.L, although Xl SFTP2.S lies at the border of R2 and R3. These proteins have the lowest κ and Ω values, which are close to zero, indicating a uniform distribution of charge throughout these proteins. They also have the highest FCR values, indicating a significant proportion (~35%) of charged residues. Such strong polyampholytes are often non-globular molecules that can adopt defined local secondary structures such as random coils and hairpins. The high proline and lysine content (20.6% and 18.9%, respectively) in Xl SFTP2.L suggests the formation of several dozen polyproline helical tracts or bends, some with distinct electrostatic properties, confirmed by results (not shown) from the PPIIPRED algorithm [92].

The catGRANULE algorithm predicts protein “foci formation” based on several criteria such as intrinsic disorder and over-representation of residues arginine, glycine, and phenylalanine, as found in a dataset of granule-forming proteins [71]. It has been employed in an assessment of functionally diverse proteins [93,94]. We took advantage of available online sequence analysis (http://s.tartaglialab.com/) to examine (accessed 21 January 2021) (Table 3) the expected phase separation of human profilaggrin and profilaggrin 2, and the four frog SFTPS. There were three prominent outcomes from this assessment. 

First is the overall “snapshot” of the six SFTPs as provided by the catGRANULE algorithm propensity scores: Hs PF1 and Hs PF2 3.553 and 4.418, respectively, and the frog SFTPs ranging from 2.019 for Xl SFTP2.S down to 0.063 for Xl SFTP1.L (Table 3). For context, catGRANULE values calculated across the human proteome [69] range from +7.808 to −8.434, with the expectations of phase separation increasing as the positive values increase. Recalling that the catGRANULE propensity scores are equivalent to +/− the number of standard deviations away from the mean [71,95,96], it is then informative to highlight that scores > +/−2 have surpassed 95% of proteins within the expected normal distribution of such scores across the proteome [69], with scores > +/−3 SD away from the mean beyond 99.7% of other values. Thus, the Hs PF1 and Hs PF2 at 3.553 and 4.418 do numerically cluster at scores apart from even the highest scoring Xl SFTP2.S at 2.019 (Table 3); however, it is important to note that this one assessment may be biased to granule expectation by the protein’s compositional bias with relatively high glutamine and threonine. 

**Figure 7:**
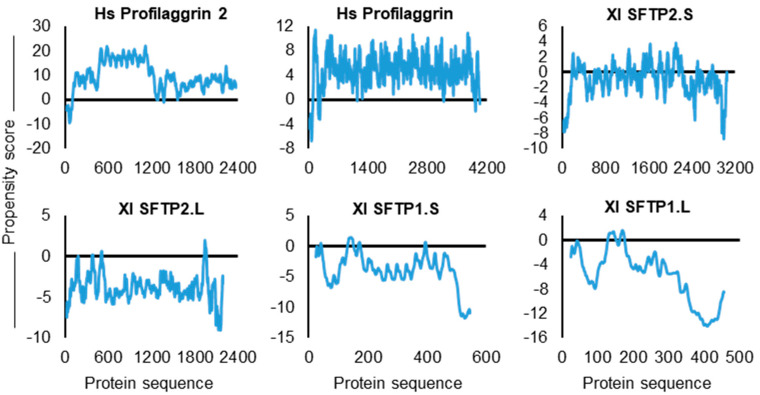
catGRANULE assessment of two human SFTPs, profilaggrin and profilaggrin 2, and four frog SFTPs. Graphs are ordered for the six proteins in decreasing overall propensity score as calculated within the algorithm and reported in Table 3. The plotted profile per protein amino acid residue does not provide predicted values for the first and last 25 amino acids of submitted sequences due to the sliding window size in algorithm calculations. Note scaling differences on the y-axis for propensity scores as well as the relative position of the zero score.

Second are the catGRANULE profiles plotted along the SFTP sequences. These graphs provide a representation of the contribution to granule propensity per amino acid residue. In viewing the algorithm-generated SFTP plots, it is important to note scaling differences on the y-axis for propensity scores as well as the relative position of the zero-score value (Figure 7). With this in mind, it is striking to note the vast majority of Hs PF1 and Hs PF2 amino acid residues above zero, but significantly fewer above zero for Xl SFTP2.S. Magnifying this downward trend are Xl SFTP2.L, Xl SFTP1.S, and Xl SFTP1.L, with almost all of the residues far below zero (Figure 7).

Third is the dramatically different occurrence (Table 3) across the six SFTPs of arginine, glycine, and phenylalanine. Within the training set for the catGRANULE algorithm, these amino acids are enriched in granule-forming proteins. For the cumulative presence of these three amino acids (Table 3), there is a conspicuous drop-off from 28.70% and 24.20% for Hs PF1 and Hs PF2, respectively, down to 4.70% to 2.30% among the four frog SFTPs. These three assessments help sort Hs PF1 and Hs PF2 versus the four frog SFTPs into two cohorts, possibly reflecting the likelihood (Hs PF1 and Hs PF2), or not (frog SFTPs), of KG formation from these related but compositionally different SFTPs.

The six SFTPs discussed here are characterized to be IDPs as per PONDR-FIT (Figure 5a). Phase separation of human profilaggrin is integral to keratohyalin granule formation [7]. Allying these results with CIDER and catGRANULE analysis may help explain the “difference” in these “similar” proteins as far as the absence of frog SFTP KG formation [57]. IDP qualities across the six proteins are retained but derived from quantitatively and qualitatively different amino acids (Figure 5d,e) which, together, impact multiple protein characteristics (Figure 6) and, ultimately, the propensity for granule formation (Figure 7 and Table 3).

Eckhart and colleagues reported that SFTPs, such as the human profilaggrins and *Xenopus* proteins presented here, had an early genesis in the EDC in the last common ancestral organism [57]. LLPS comparison of these and additional EDC paralogues within, and orthologues across, species will require future study. Nevertheless, despite the divergence of the amino acid identity, what does seem to have originated with any in-common ancestral sequence and been retained by extant SFTPs is their residue content preference for those conferring intrinsic disorder. This trait across the duplication and diversification in SFTPs suggests disorder has been at least compatible with their differentiation-associated functions, even if not driving the phase separation of all SFTPs into observable KG. Our evaluation here suggests SFTPs as examples of evolutionary flexible disorder, where disorder is conserved, but the amino acid residues providing it have nevertheless diverged [97,98]. Resolution of the LLPS potential for other SFTPs, such as those expressed in amphibian, avian, and reptilian species in the absence of reported KG, will require additional assessment including, but likely not limited to, factors affecting LLPS of other proteins such as [99] post-translational modification, tendency for self-interaction, subcellular local pH and other ions, and concentration and relative size of the SFTP [7,84,100,101].

## 3. Methodology

### 3.1. Literature Inquiry 

We queried PubMed (https://pubmed.ncbi.nlm.nih.gov/ accessed on 26 April 2021) as a database for publications relevant to overlapping fields of keratinocyte biology and intrinsic disorder. Search parameters are provided in the legend for Table 1, and returned publications were manually reviewed to remove coincidental hits.

### 3.2. Bioinformatics Assessments 

Note that for better distinction in plot legends, we referred to the two human SFTPs as profilaggrin 1 (UniProt P20930) and profilaggrin 2 (UniProt Q5D862), although most reports omit the number designation in mentioning the prototypical protein profilaggrin 1. *Gallus gallus* loricrins are from previously reported supplementary data files [43]. In silico analyses of proteins were conducted using amino acid sequences from the indicated UniProt (https://www.uniprot.org/) accession number (accessed 13 January 2021) or from sequences in cited publications. Profiling of intrinsic disorder along a protein sequence was performed with PONDR-FIT [56] to take advantage of its meta-predictor design inclusive of its six-component algorithms. Global PONDR-FIT scores refer to an average of individual residue scores returned from online analysis (http://original.disprot.org/pondr-fit.php) (accessed 21 January 2021). CH and CDH analyses were performed at (http://www.pondr.com/) (accessed 29 January 2021) [102,103] as previously presented [10]. Amino acid compositional bias was determined at (http://www.cprofiler.org/) (accessed 29 January 2021) as described [64]. Amino acid counts, molecular weight (MW), and other characteristics reported in Table 2 were calculated at https://web.expasy.org/protparam/ (accessed 5 February 2021).

Propensity toward foci formation was gauged by the catGRANULE algorithm [71] (accessed 21 January 2021) available online (http://s.tartaglialab.com/) for an indication of the assessed proteins’ possible liquid–liquid phase separation. Utility of the catGRANULE algorithm has been reported across a wide spectrum of proteins [93,94]. Regarding the tendency for a protein to undergo LLPS, catGRANULE assesses protein length and overall amino acid composition, including arginine, glycine, phenylalanine proportion, conformational disorder, and several other physicochemical properties [69,71]. Submitted sequences return a relative predisposition, or propensity score, for the entire protein regarding phase separation. A previous human proteome-wide use of catGRANULE reported values from a minimum of −8.434 to a maximum of +7.808 [69], with increasing positive scores reflecting increasing tendency to phase separate. Importantly, these values represent the number of standard deviations (SD) away from the mean [71,95,96]. For example, scores equal to or greater than +/−3 SD away from the mean have surpassed 99.7% of other values [69]. We provide further consideration for the magnitude of catGRANULE propensity scores in Section 2 when returned scores for individual proteins are presented. catGRANULE and LARKS scores were also retrieved from supplementary files of a previously reported proteome analysis [69].

The Das–Pappu diagram of states [90], as well as the values in (Figure 6) describing the proposed structural conformation of human profilaggrin 1 and profilaggrin 2 and the four frog SFTPs, was derived by submitting sequences to Classification of Intrinsically Disordered Ensemble Relationships or the CIDER web server (http://pappulab.wustl.edu/CIDER/analysis/) [91] (accessed 6 April 2021). The reported values include κ, which specifies the patterning of oppositely charged residues, 0 representing a protein where such residues are well dispersed to 1 indicating segregation. Analogous to this is Ω quantifying the distribution of prolines, again ranging from 0 to 1. FCR and NCPR denote the fraction of charged residues and net charge per residue, respectively. The average hydropathy scores across the length of the protein were based on the well-accepted Kyte–Doolittle scale. Lastly, the diagram of states classification, where the fraction positive (*f+*) and fraction negative (*f*−) values calculated for each protein were replotted using GraphPad Prism ver. 9.1.0, considers the charge–hydropathy relationships in these SFTPs and, in doing so, classifies them into five conformational categories designated on the plot. 

## 4. Conclusions

### 4.1. Protein ID in the Keratinocyte Proteome Facilitates Cell Function

Our data extraction from the literature (e.g., loricrin and LARKS) and de novo analysis conducted here (e.g., SFTPs with PONDR-FIT, CIDER, and catGRANULE) clearly position the keratinocyte proteome as a highly promising but mostly untapped reservoir of additions to ID conformation studies for LLPS and membraneless organelles. The discovery potential with these cells is significant considering that of >21,000 human protein sequences previously assessed via catGRANULE [69], it was several keratinocyte-expressed proteins that returned the highest propensity scores. This includes the SFTPs hornerin (5.572), profilaggrin 2 (4.418), and profilaggrin (3.553), placing them well within the top 0.5% of all scored proteins. Finally, we note it was loricrin, the keratinocyte cornified envelope protein participating in granule formation distinct (see above) from SFTP-based KG, which had a catGRANULE propensity score of 7.808, the highest across the human proteome [69].

Generation and maintenance of a protective barrier function can be considered the raison d’être of the epidermis across diverse terrestrial and aquatic vertebrate species. Proteins encoded by the EDC, and likely other keratinocyte IDPs, could prove to be a revealing laboratory for investigating intrinsic disorder, liquid–liquid phase separation, and membraneless organelles. How “biological copolymers” have been “evolutionarily edited” [104] to meet the genesis of epidermal keratinocyte subcellular structures, including, but not necessarily limited to, KG and CE, would be an adventure in wonderland indeed. Nevertheless, computational and in vitro assessments of proteins consistent with their phase separation in cells will still have significant in vivo testing required, as stressed by Tjian and colleagues [105], to assure that the observed protein condensates are indeed occurring from local supersaturation and liquid–liquid demixing, and that results can be compared across systems, as emphasized by Pansca and coworkers [99].

### 4.2. Investigations of Protein ID Synergize with Keratinocyte Biology

- Despite the few reports to date, the importance of IDPs in cutaneous biology can be expected to be the rule, not the exception. Intrinsic disorder is likely integral to the conformation and function not only of numerous endogenous keratinocyte proteins but also in therapeutically counteracting biofilm proteins of skin bacteria, e.g., *Staphylococcus epidermidis*, and oncogenic proteins of keratinocyte-tropic papilloma virus [25,29].
- Keratinocyte IDPs and proteins with extensive IDR, especially in upper epidermal strata, may be particularly favored in these superficial cells. Typical IDP qualities of being minimally affected (i.e., denatured) by harsh conditions or possessing “conformational plasticity” [9] could lend to understanding the resiliency of these cells as they are subjected to varying surface environmental assaults.
- The ID derived from numerous repeats enriched for disorder-promoting amino acids within keratinocyte-specific proteins may advantageously contribute to increased mammalian SFTP proteolytic sensitivity, efficiently yielding antimicrobial and hydrative peptides, respectively, from hornerin and filaggrin [5,8], and might be harnessed for clinical benefit by purposefully regulating the breakdown.
- Learning from human profilaggrin protein liquid–liquid phase separation for KG, it is worth conducting future direct biophysical experimentation with other keratinocyte-manifested structures such as cornified envelopes in the context of proteinaceous membraneless organelles (PMLO). This is especially appropriate in light of CE involucrin and loricrin self–self-protein interactions and their repeating amino acid motifs [74], which are biomolecular condensate- and PMLO-germane characteristics [75]. Strengthening this rationale is loricrin’s very favorable, pro-granule scoring in the LARKS and catGRANULE analysis. Investigation of keratinocyte sheath-like CE could add to the granule, speckle, and droplet categories of PMLO.
- Approximately 30 years of intense and elegant structural biology investigations of epithelial proteins such as keratins ([106] for review) have yielded a tremendous understanding regarding their function in tissue health and multiple disease states. We must now also face the fact that about one half of all eukaryotic proteins reside in a “dark proteome” [107] where conformational studies are often thwarted because of ID. As with Alice, we must reconsider what we think we know. It stands to reason that many keratinocyte-specific and -expressed proteins under current investigation, along with those of more established structure–function relationships, might be newly and revealingly viewed through the looking glass of ID. In this regard, keratinocyte normal physiology and pathophysiology can also be greatly affected by more broadly expressed IDPs, such as TNIP1, a repressor of inflammatory signaling, as we recently reported [53,108]. Additionally, there are the new lessons that the unique proteins of epidermal keratinocytes might provide to the IDP field. An integrated approach recently reviewed by Fuxreiter and colleagues [109] for membraneless organelles, protein–protein interaction, and phase separation in neurodegenerative disorders provides a roadmap for combining and applying ID computational and biophysical methodologies to other cell types. Such investigations of keratinocyte proteins could be a sea change moment for conformational understanding and subsequent translational cutaneous health benefits.

## Figures and Tables

**Table 1:** Search hits (primary reports and reviews) presented in reverse chronological order. PubMed search parameters: conducted on 26 April 2021 with [(skin OR cutaneous OR epiderm* OR keratinocyte) AND (“unstructured protein” OR “intrinsic disorder” OR “intrinsically disordered”)]. Unless otherwise noted (e.g., frog, squid, bacterial), cited proteins are from human samples. Intrinsic AND disorder, as separate words with the “AND” operator, were not used because they did not distinguish hits on intrinsic disease states from protein conformation references. Returned publications were manually reviewed by text searching for how the keywords appeared in the publication to remove these coincidental hits. Inclusion of “unstructured” alone produced excess off-target hits such as “unstructured interviews”, “unstructured content”, and “unstructured review”. We consider the table’s hits representational, if not all-inclusive, and apologize to any researchers not included due to the search parameters or who have published in journals not indexed by MEDLINE. The term “intrinsically disordered” alone returned 5654 PubMed hits. Abbreviations: AF-1, activating function 1; BP180, bullous pemphigoid; C, carboxyl; N, amino; ECM, extracellular matrix; EGF-R, epidermal growth factor receptor; HPV, human papilloma virus; TB, tuberculosis.

1st Author	Journal Citation	Proteins Reported as IDPs or with IDR
Tuusa, J.	[6]	hemidesmosome protein BP180
Garcia Quiroz, F.	[7]	filaggrin in keratohyalin granules
Shamilov, R.	[10]	nuclear receptor N-terminal AF-1
Levenson, R.	[11]	squid skin light-reflecting proteins
Latendorf, T.	[5]	hornerin antimicrobial peptides
Kurvits, L.	[12]	serum amyloid in skin biopsies
Okamoto, K.	[13]	C-terminus of EGF-R
Moens, M.	[14]	avian skin viral protein
Gopalan, A.	[15]	TB proteins in skin lesions
Uversky, V.	[9]	biofilms of pathogenic *Staphylococcus epidermidis*
Singh, I.	[16]	C-terminus of EGF-R
Rauscher, S.	[17]	skin ECM protein elastin
Yarawsky, A.	[18]	biofilms of pathogenic *Staphylococcus epidermidis*
Keppel, T.	[19]	C-terminus of EGF-R
Muiznieks, L.	[20]	skin ECM protein elastin
Levenson, R.	[21]	squid skin light-reflecting proteins
Wang, B.	[22]	ubiquitin-conjugating enzyme
Bray, D.	[23]	keratin N- and C-termini
Kornreich, M.	[24]	keratin N- and C-termini
Whelan, F.	[25]	biofilms of pathogenic *Staphylococcus epidermidis*
Mukherjee, S.	[26]	proteins of cutaneous pathogen *Leishmania major*
Joseph, S.	[27]	DNA-binding protein
Richer, B.	[28]	skin ECM collagen subdomain
Xue, B.	[29]	HPV oncoproteins
Yates, C.	[30]	C-terminus of EGF-R
Scorciapino, M.	[31]	frog skin antibacterial peptide
Akinshina, A.	[32]	keratin N- and C-termini
Graham, L.	[33]	frog skin-secreted adhesive protein
Lewitzky, M.	[34]	C-terminus of EGF-R
Shan, Y.	[35]	C-terminus of EGF-R
Rauscher, S.	[36]	skin ECM protein elastin
Lehoux, M.	[37]	HPV oncoproteins
Majczak, G.	[38]	antimicrobial dermicidin protein
Uversky, V.	[39]	HPV oncoproteins

**Table 2:** SFTP characteristics across species. Sequences were submitted to https://web.expasy.org/protparam/ and http://original.disprot.org/pondr-fit.php (accessed 5 February 2021). Arginine bias: calculated as ARG/[ARG + LYS]. PONDR-FIT scores are averaged across the sequence entire length, including the likely structured S100-type amino terminus.

Trait	Hs PF1	Hs PF2	Xl SFTP2.S	Xl SFTP2.L	Xl SFTP1.S	Xl SFTP1.L
Sequence source	UniProt P20930	UniProt Q5D862	GenBank: OCT66701.1	GenBank: OCT69537.1 [57]	[57]	GenBank: XP_018087213.1
# AA	4061	2391	3075	2220	570	480
MW	435 kDa	248 kDa	349 kDa	249 kDa	64 kDa	54 kDa
Theoretical pI	9.24	8.45	8.77	8.03	8.65	6.90
ARG bias	0.885	0.824	0.031	0.033	0.012	0.036
ARG %	10.8	6.1%	0.60	0.60	0.2%	0.4%
LYS %	1.40	1.3%	17.20	18.30	15.3%	11.2%
HIS %	10.20	10.3	1.30	4.80	1.4%	2.7%
PONDR-FIT score w/N-term	0.895	0.896	0.852	0.740	0.722	0.705
Aliphatic index	19.76	15.97	15.62	26.91	62.39	49.65

**Table 3:** catGRANULE overall protein propensity score, and Arg, Gly, and Phe occurrence for two human SFTPs, profilaggrin and profilaggrin2, and four frog SFTPs. Amino acid percentages were determined with https://web.expasy.org/protparam/ (accessed (21 January 2021).

SFTP	Propensity Score	Gly	Arg	Phe	SUM: Gly, Arg, Phe
Hs PF2	4.418	20.30%	6.10%	2.30%	28.70%
Hs PF1	3.553	12.80%	10.80%	0.60%	24.20%
Xl SFTP2.S	2.019	3.50%	0.60%	0.60%	4.70%
Xl SFTP2.L	1.179	0.80%	0.60%	0.90%	2.30%
Xl SFTP1.S	0.561	2.50%	0.20%	0.70%	3.40%
Xl SFTP1.L	0.063	2.50%	0.40%	1.50%	4.40%

## Data Availability

The data presented in this study are available within the article.
